# Structural data of phenanthrene-9,10-dicarbonitriles

**DOI:** 10.1016/j.dib.2019.104605

**Published:** 2019-10-05

**Authors:** Anastasiia M. Afanasenko, Alexander S. Novikov, Tatiana G. Chulkova, Yakov M. Grigoriev, Ilya E. Kolesnikov, Stanislav I. Selivanov, Galina L. Starova, Andrey A. Zolotarev, Anatoly N. Vereshchagin, Michail N. Elinson

**Affiliations:** a Saint Petersburg State University, 7/9 Universitetskaya Nab., Saint Petersburg, 199034, Russia; b Stratingh Institute for Chemistry, University of Groningen, Nijenborgh 4, 9747 AG, Groningen, the Netherlands; c N. D. Zelinsky Institute of Organic Chemistry, 47 Leninsky Prospect, Moscow, 119991, Russia

**Keywords:** Aromatic stacking interactions, Non-covalent interactions, Phenanthrene-9,10-dicarbonitriles, Single-crystal X-ray diffraction

## Abstract

In this data article, we present the single-crystal XRD data of phenanthrene-9,10-dicarbonitriles. Detailed structure analysis and photophysical properties were discussed in our previous study, “Intermolecular interactions-photophysical properties relationships in phenanthrene-9,10-dicarbonitrile assemblies” (Afanasenko et al., 2020). The data include the intra- and intermolecular bond lengths and angles.

**Table:** Specifications Table

Subject	Chemistry
Specific subject area	Single crystal data of phenanthrene-9,10-dicarbonitriles
Type of data	Table Figure
How data were acquired	Single-crystal XRD: The X-ray diffraction data were collected on an Agilent Technologies Excalibur Eos and Supernova Atlas diffractometers. The structures have been solved by the direct methods and refined by means of the SHELXL–97 program incorporated in the OLEX2 program package.
Data format	Raw and Analyzed
Parameters for data collection	Crystals were analysed at a temperature of 100 K. Single crystal X-ray diffraction data was collected with CuKα (3,6-difluorophenanthrene-9,10-dicarbonitrile, 3,6-dimethylphenanthrene-9,10-dicarbonitrile) and MoKα (3,6-dimethoxyphenanthrene-9,10-dicarbonitrile) radiation.
Description of data collection	Crystals of compounds were immersed in cryo-oil, mounted in a nylon loop.
Data source location	Saint Petersburg State University, Saint Petersburg, Russia
Data accessibility	Crystal data have been deposited at the Cambridge Crystallographic Data Centre (CCDC) with deposition numbers CCDC 1821025, ССDC 1821026, and ССDC 1820117 (http://www.ccdc.cam.ac.uk/conts/retrieving.html, e-mail: deposit@ccdc.cam.ac.uk). With the article
Related research article	Anastasiia M. Afanasenko, Alexander S. Novikov, Tatiana G. Chulkova, Yakov M. Grigoriev, Ilya E. Kolesnikov, Stanislav I. Selivanov, Galina L. Starova, Andrey A. Zolotarev, Anatoly N. Vereshchagin, Michail N. Elinson. Intermolecular interactions-photophysical properties relationships in phenanthrene-9,10-dicarbonitrile assemblies. J. Mol. Struct. 1199 (2020) 126789, https://doi.org/10.1016/j.molstruc.2019.07.036.

**Table:** 

**Value of the Data** • This data would be valuable for other properties studies of phenanthrene-9,10-dicarbonitriles. • The data in this article will be useful for researchers who study non-covalent interactions. • This data provide a new strategy to control the association pattern in the crystal state.

## 1 Data

**Fig. 1:**
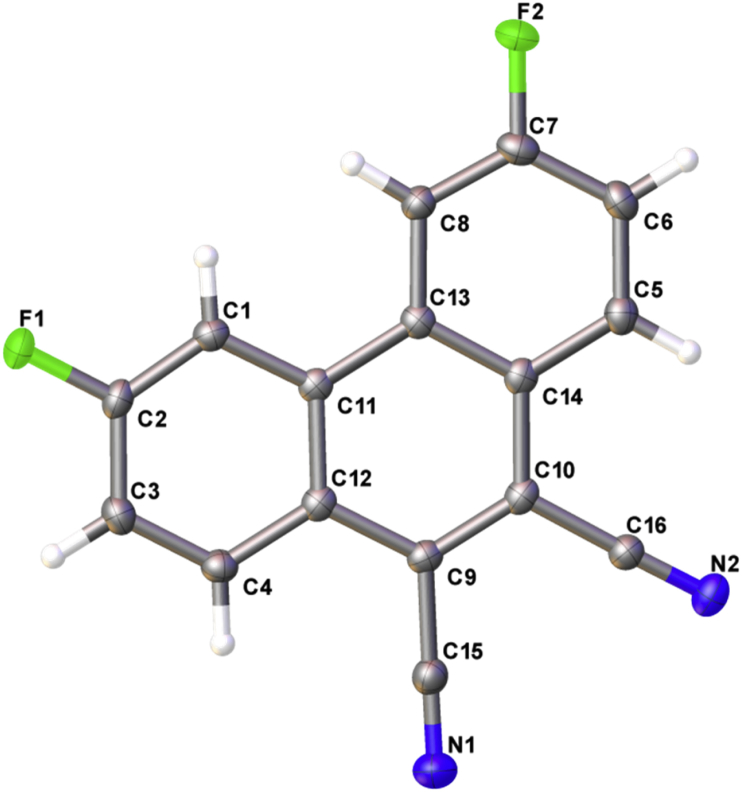
Structure of 3,6-difluorophenanthrene-9,10-dicarbonitrile.

**Fig. 2:**
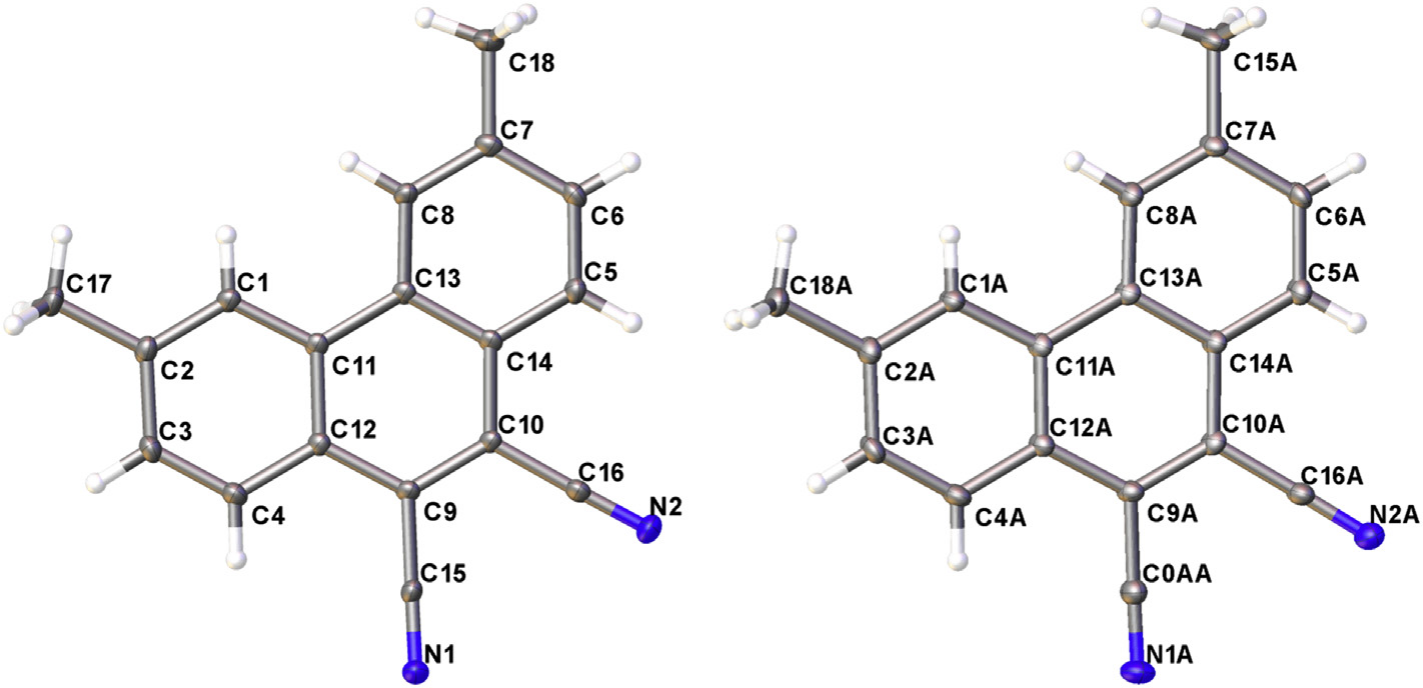
Structure of 3,6-dimethylphenanthrene-9,10-dicarbonitrile.

**Fig. 3:**
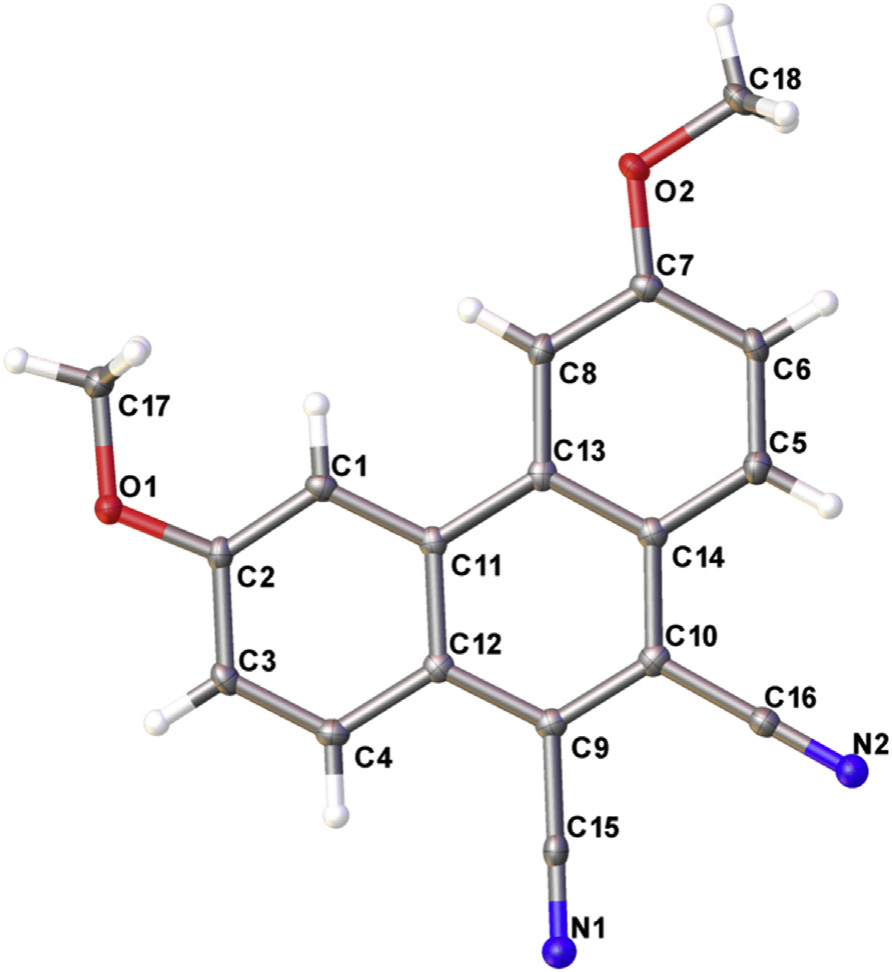
Structure of 3,6-dimethoxyphenanthrene-9,10-dicarbonitrile.

**Fig. 4:**
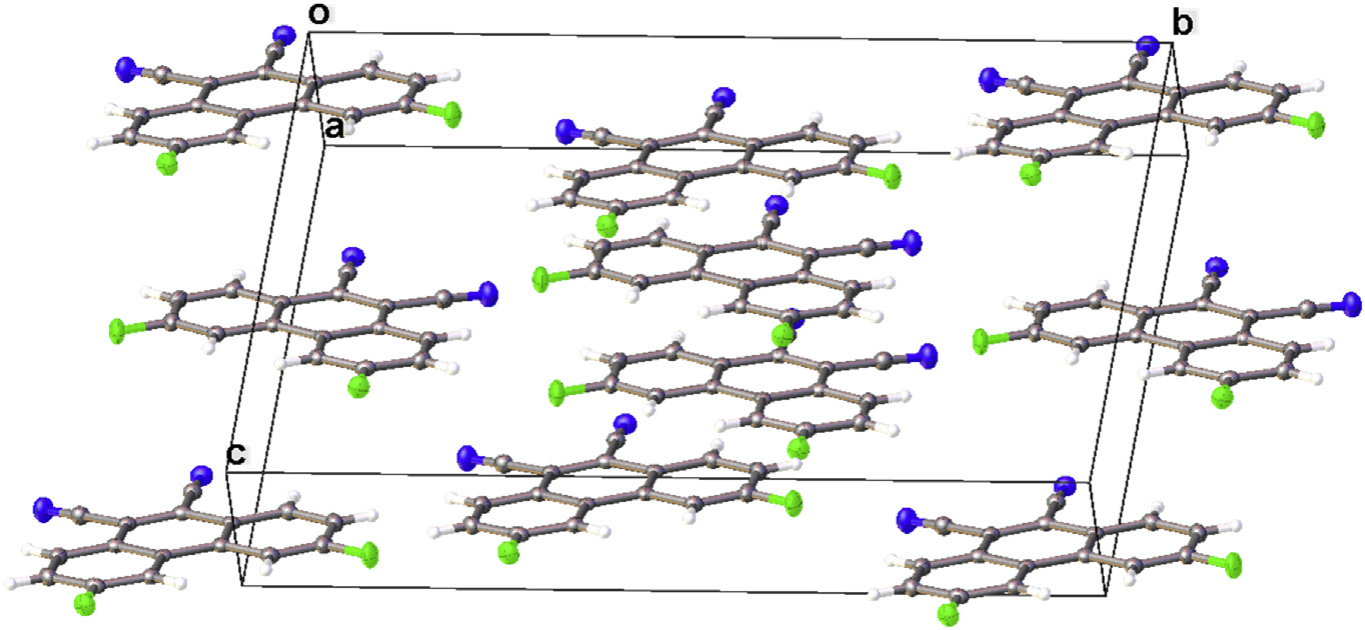
The crystal packing of 3,6-difluorophenanthrene-9,10-dicarbonitrile.

**Fig. 5:**
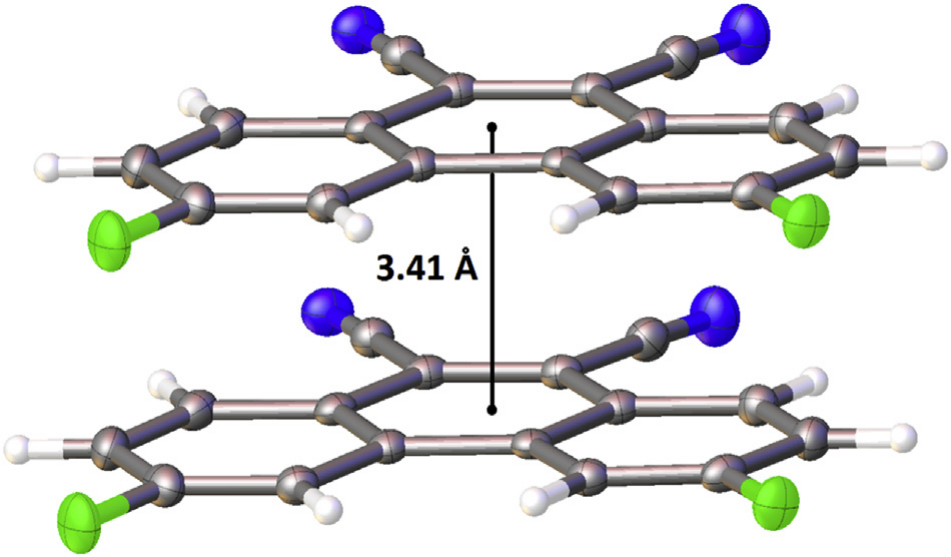
π-Stacking in the crystal structure of 3,6-difluorophenanthrene-9,10-dicarbonitrile.

**Fig. 6:**
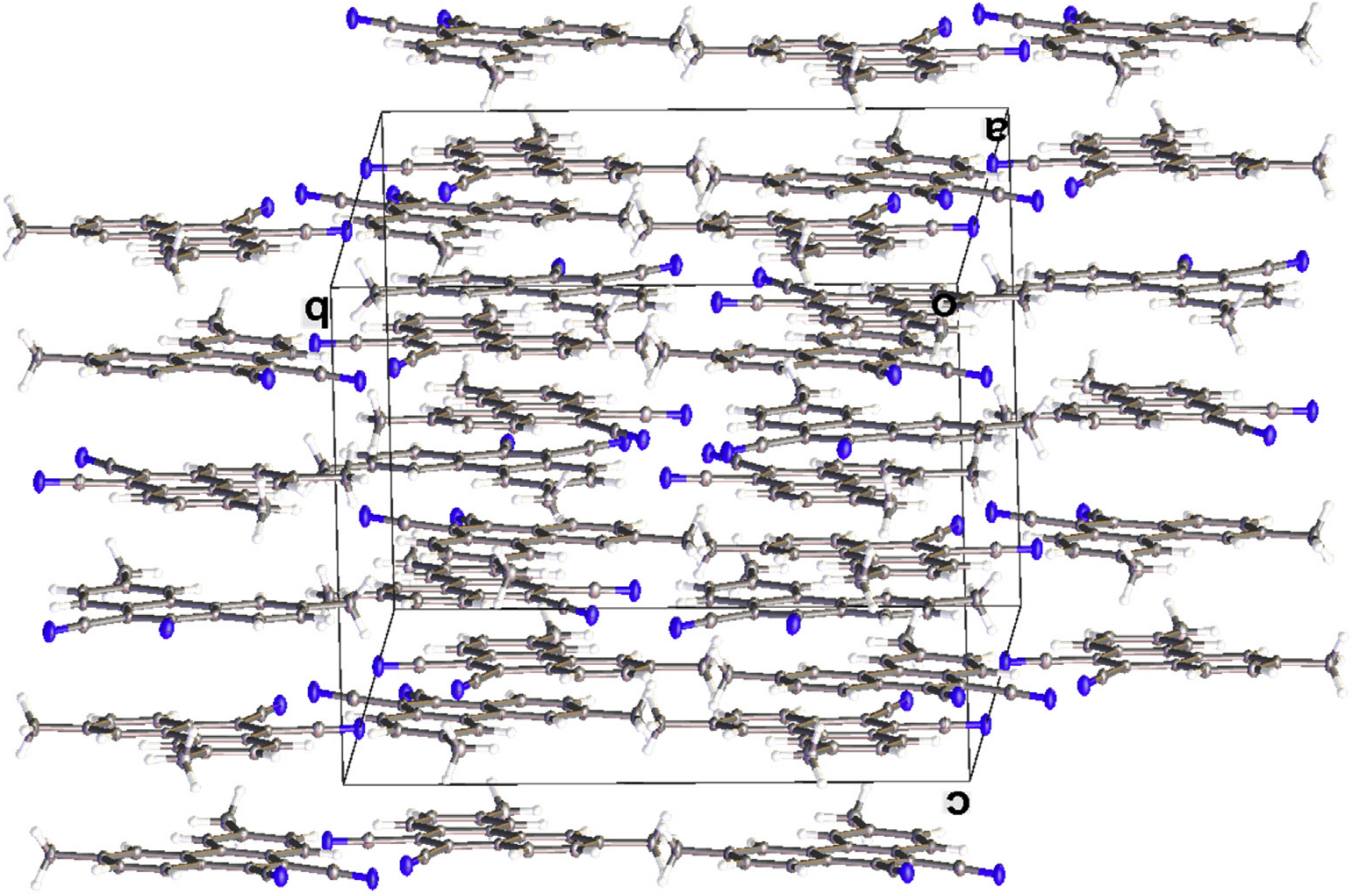
The crystal packing of 3,6-dimethylphenanthrene-9,10-dicarbonitrile.

**Fig. 7:**
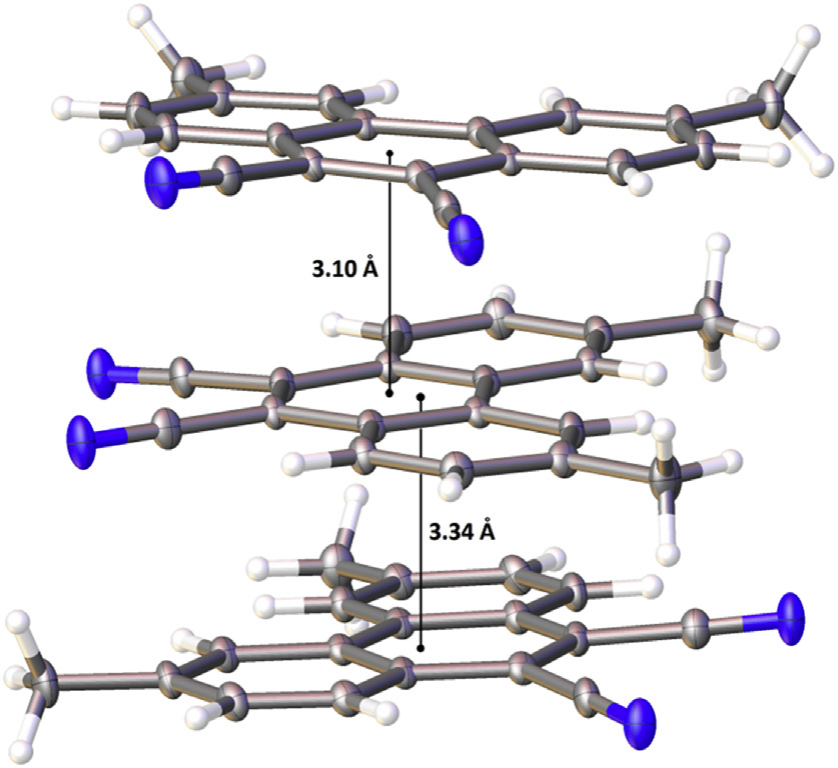
π-Stacking in the crystal structure of 3,6-dimethylphenanthrene-9,10-dicarbonitrile.

**Fig. 8:**
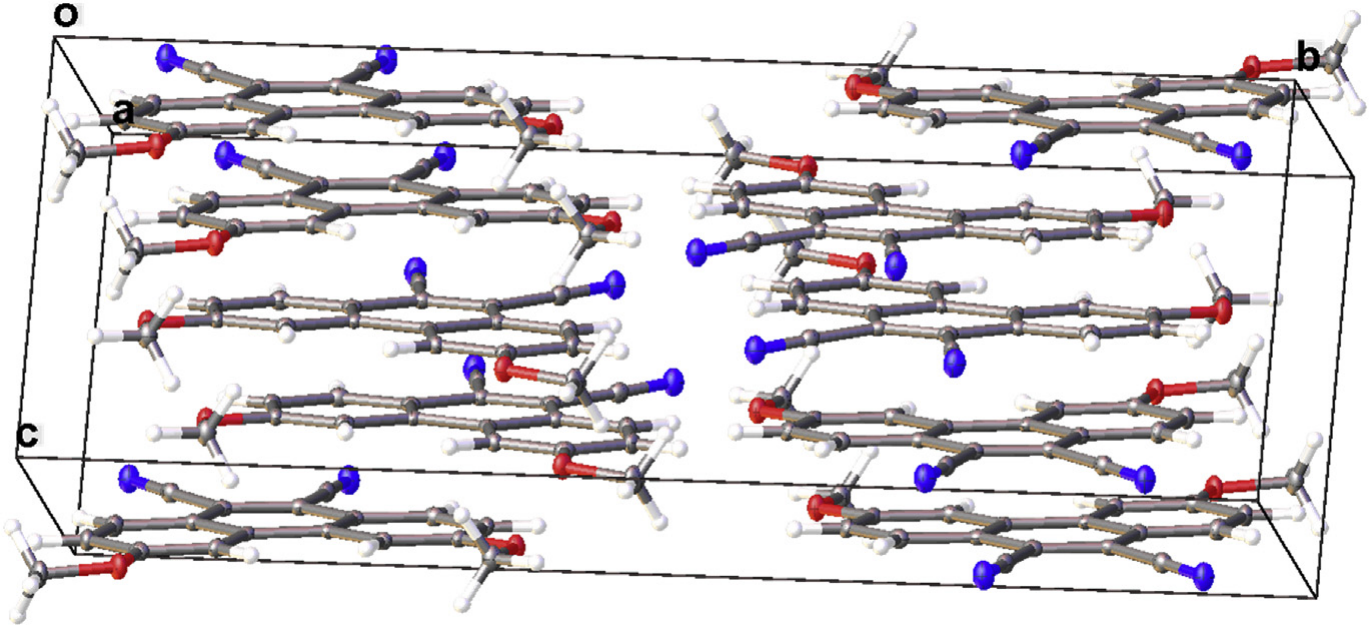
The crystal packing of 3,6-dimethoxyphenanthrene-9,10-dicarbonitrile.

**Fig. 9:**
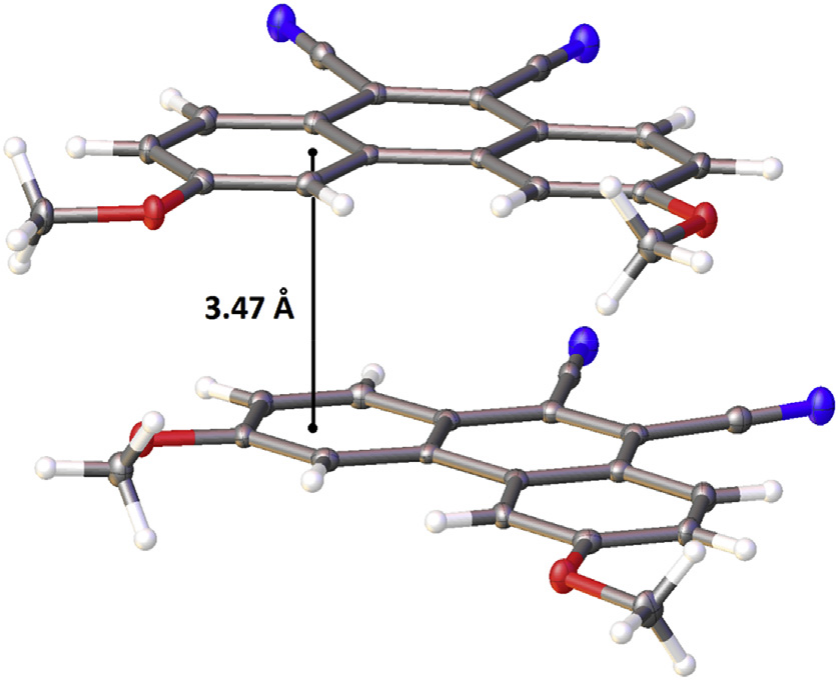
π-Stacking in the crystal structure of 3,6-dimethoxyphenanthrene-9,10-dicarbonitrile.

In this article, the X-ray information for 3,6-difluorophenanthrene-9,10-dicarbonitrile, 3,6-dimethylphenanthrene-9,10-dicarbonitrile, and 3,6-dimethoxyphenanthrene-9,10-dicarbonitrile is represented. The structures of phenanthrene-9,10-dicarbonitriles are shown in Fig. 1, Fig. 2, Fig. 3. Fractional atomic coordinates and equivalent isotropic displacement parameters for 3,6-difluorophenanthrene-9,10-dicarbonitrile, 3,6-dimethylphenanthrene-9,10-dicarbonitrile, and 3,6-dimethoxyphenanthrene-9,10-dicarbonitrile are listed in Table 1, Table 2, and Table 3, respectively. The bond lengths, angles, and torsion angles for phenanthrene-9,10-dicarbonitriles are listed in Table 4, Table 5, Table 6, Table 7, Table 8, Table 9, Table 10, Table 11, Table 12. The crystal packing of phenanthrene-9,10-dicarbonitriles is shown in Fig. 4, Fig. 6, Fig. 8. Information about π-stacking for phenanthrene-9,10-dicarbonitriles is presented in Fig. 5, Fig. 7, Fig. 9. The data in Table 13, Table 14, Table 15 show the intermolecular distances for phenanthrene-9,10-dicarbonitriles.

**Table 1:** Fractional atomic coordinates (×10^4^) and equivalent isotropic displacement parameters (Å^2^ × 10^3^) for 3,6-difluorophenanthrene-9,10-dicarbonitrile. U_eq_ is defined as 1/3 of the trace of the orthogonalised U_IJ_ tensor.

Atom	x	y	z	U (eq)
F1	5032 (4)	6348.1 (6)	8033.0 (9)	24.5 (3)
F2	8212 (4)	3296.2 (6)	6013.3 (9)	29.0 (3)
C2	6390 (6)	6326.4 (11)	7253.2 (13)	18.7 (5)
C14	10137 (6)	5050.9 (11)	4824.8 (14)	17.0 (5)
C8	8202 (6)	4447 (1)	6053.4 (14)	18.1 (5)
C11	8250 (5)	5687.3 (10)	6074.6 (13)	15.6 (5)
N2	13427 (6)	5638.6 (9)	2923.6 (13)	25.2 (4)
C4	8453 (6)	6900.1 (10)	6064.8 (14)	19.2 (5)
C9	10378 (5)	6260.3 (11)	4819.5 (13)	17.0 (5)
C6	10079 (6)	3856.3 (10)	4805.0 (14)	22.0 (5)
C13	8857 (6)	5060.3 (11)	5656.8 (13)	16.9 (5)
N1	12149 (6)	7336.7 (9)	4065.6 (14)	31.2 (5)
C16	12274 (6)	5658.6 (10)	3583.9 (13)	18.8 (5)
C3	7151 (6)	6923.7 (11)	6862.3 (14)	21.1 (5)
C15	11317 (6)	6864.7 (11)	4397.5 (14)	21.3 (5)
C1	6897 (6)	5723.0 (11)	6893.2 (14)	17.8 (5)
C12	9010 (5)	6288.3 (11)	5658.6 (13)	17.4 (5)
C7	8849 (6)	3878.4 (11)	5621.8 (14)	22.0 (5)
C5	10711 (6)	4444 (1)	4409.5 (14)	20.1 (5)
C10	10884 (6)	5669.2 (10)	4421.3 (13)	17.6 (5)

**Table 2:** Fractional atomic coordinates (×10^4^) and equivalent isotropic displacement parameters (Å^2^ × 10^3^) for 3,6-dimethylphenanthrene-9,10-dicarbonitrile. U_eq_ is defined as 1/3 of the trace of the orthogonalised U_IJ_ tensor.

Atom	*x*	*y*	*z*	U (eq)
N1	−82.7 (11)	4030.6 (6)	3353.3 (9)	22.7 (3)
N2	3096.7 (11)	5002.8 (7)	4957 (1)	25.8 (3)
N1A	4515.2 (12)	5029.2 (7)	8469.4 (11)	27.2 (3)
N2A	6832.0 (12)	3359.2 (7)	9233.7 (10)	25.4 (3)
C14	4028.7 (11)	3116.8 (7)	5481.9 (9)	13.5 (3)
C13	3954.8 (11)	2321.8 (7)	5456.0 (9)	13.3 (3)
C14A	4025.3 (11)	2335.2 (7)	7989.6 (9)	13.9 (3)
C10	2926.2 (11)	3548.2 (7)	4936.7 (9)	13.7 (3)
C12A	2366.9 (12)	3591.6 (7)	7243.0 (9)	14.7 (3)
C13A	2743.0 (12)	2209.4 (7)	7371.7 (9)	14.0 (3)
C11	2756.8 (11)	1951.2 (7)	4904.0 (9)	13.3 (3)
C11A	1910.4 (12)	2851.1 (7)	6978.5 (9)	13.9 (3)
C9A	3658.6 (12)	3694.6 (7)	7885.3 (9)	14.9 (3)
C12	1685.3 (11)	2394.2 (7)	4381.8 (9)	13.6 (3)
C4A	1561.1 (12)	4214.9 (7)	6859.8 (10)	17.4 (3)
C2	1485.8 (12)	812.0 (7)	4344.1 (9)	16.5 (3)
C3A	342.4 (13)	4106.0 (7)	6219 (1)	18.7 (3)
C10A	4463.0 (11)	3092.9 (7)	8222.5 (9)	14.9 (3)
C1	2623.3 (12)	1161.5 (7)	4865.2 (9)	15.1 (3)
C6A	4397.2 (12)	992.0 (7)	8146.7 (10)	17.5 (3)
C7A	3131.3 (12)	855.0 (7)	7526.7 (10)	17.0 (3)
C0AA	4128.1 (12)	4439.5 (7)	8195.5 (10)	18.6 (3)
C15	748.9 (12)	3658.0 (7)	3822.9 (10)	16.6 (3)
C1A	645.1 (12)	2763.4 (7)	6325.9 (9)	15.3 (3)
C5A	4832.2 (12)	1711.3 (7)	8369.6 (10)	16.7 (3)
C2A	−133.3 (12)	3374.6 (7)	5937 (1)	16.8 (3)
C9	1803.5 (11)	3199.0 (7)	4397.2 (9)	14.0 (3)
C8A	2337.8 (12)	1460.1 (7)	7154.6 (9)	15.9 (3)
C16	3009.0 (11)	4356.8 (8)	4942.2 (10)	17.6 (3)
C7	6192.6 (12)	2264.9 (7)	6493.5 (9)	17.1 (3)
C5	5183.5 (12)	3477.2 (7)	6021.4 (10)	16.5 (3)
C16A	5777.6 (12)	3233.9 (7)	8800.7 (10)	17.6 (3)
C8	5063.2 (12)	1911.6 (7)	5967.0 (9)	15.2 (3)
C4	519.7 (12)	2034.8 (7)	3859.7 (10)	17.2 (3)
C17	1375.5 (13)	−33.5 (7)	4287.7 (11)	21.8 (3)
C6	6239.4 (12)	3058.7 (8)	6520.8 (9)	17.4 (3)
C3	421.5 (12)	1268.5 (8)	3844.7 (10)	18.8 (3)
C15A	2653.5 (14)	62.2 (7)	7283.1 (11)	23.2 (3)
C18A	−1455.5 (12)	3268.0 (8)	5208.6 (11)	22.0 (3)
C18	7359.6 (13)	1813.5 (8)	7031.6 (11)	25.2 (3)

**Table 3:** Fractional atomic coordinates (×10^4^) and equivalent isotropic displacement parameters (Å^2^ × 10^3^) for 3,6-dimethoxyphenanthrene-9,10-dicarbonitrile. U_eq_ is defined as 1/3 of the trace of the orthogonalised U_IJ_ tensor.

Atom	*x*	*y*	*z*	U (eq)
C1	9340 (2)	7965.2 (7)	3471 (2)	12.7 (4)
C2	8802 (2)	8553.7 (7)	3703 (2)	12.5 (4)
C3	7248 (2)	8670.0 (7)	4359 (2)	13.7 (4)
C4	6265 (2)	8196.0 (8)	4780 (2)	13.7 (4)
C5	8313 (2)	5746.8 (7)	3835 (2)	15.2 (4)
C6	9789 (2)	5618.4 (7)	3145 (2)	14.8 (4)
C7	10816 (2)	6098.3 (7)	2686 (2)	13.6 (4)
C8	10357 (2)	6697.4 (7)	2934 (2)	12.8 (3)
C9	5764 (2)	7083.5 (7)	5013 (2)	12.6 (3)
C10	6262 (2)	6491.2 (7)	4808 (2)	13.1 (4)
C11	8336 (2)	7468.7 (7)	3890 (2)	11.2 (3)
C12	6783 (2)	7585.2 (7)	4561 (2)	12.0 (3)
C13	8852 (2)	6838.9 (7)	3638 (2)	12.0 (4)
C14	7810 (2)	6353.4 (7)	4096 (2)	12.7 (4)
C15	4209 (2)	7206.7 (7)	5739 (2)	13.9 (4)
C16	5235 (2)	6005.2 (7)	5362 (2)	14.0 (4)
C17	11302 (2)	8964.3 (8)	2703 (2)	17.3 (4)
C18	12774 (2)	5410.8 (8)	1616 (2)	19.5 (4)
N1	2976.3 (19)	7306.4 (7)	6322 (2)	19.8 (3)
N2	4438.9 (19)	5619.1 (7)	5838 (2)	19.1 (3)
O1	9690.6 (15)	9060.9 (5)	3343.7 (15)	16.0 (3)
O2	12295.8 (15)	6021.9 (5)	1975.2 (15)	16.1 (3)

**Table 4:** Bond lengths for 3,6-difluorophenanthrene-9,10-dicarbonitrile.

Atom	Atom	Length/Å
F1	C2	1.354 (2)
F2	C7	1.357 (3)
C2	C3	1.393 (3)
C2	C1	1.364 (3)
C14	C13	1.417 (3)
C14	C5	1.413 (3)
C14	C10	1.438 (3)
C8	C13	1.417 (3)
C8	C7	1.365 (3)
C11	C13	1.453 (3)
C11	C1	1.411 (3)
C11	C12	1.418 (3)
N2	C16	1.147 (3)
C4	C3	1.371 (3)
C4	C12	1.414 (3)
C9	C15	1.446 (3)
C9	C12	1.442 (3)
C9	C10	1.368 (3)
C6	C7	1.388 (3)
C6	C5	1.369 (3)
N1	C15	1.141 (3)
C16	C10	1.442 (3)

**Table 5:** Bond angles for 3,6-difluorophenanthrene-9,10-dicarbonitrile.

Atom	Atom	Atom	Angle/˚
F1	C2	C3	117.92 (18)
F1	C2	C1	118.19 (19)
C1	C2	C3	123.9 (2)
C13	C14	C10	118.7 (2)
C5	C14	C13	120.3 (2)
C5	C14	C10	121.0 (2)
C7	C8	C13	118.74 (19)
C1	C11	C13	122.00 (18)
C1	C11	C12	117.92 (18)
C12	C11	C13	120.08 (18)
C3	C4	C12	120.78 (19)
C12	C9	C15	119.7 (2)
C10	C9	C15	119.1 (2)
C10	C9	C12	121.2 (2)
C5	C6	C7	117.8 (2)
C14	C13	C11	119.8 (2)
C8	C13	C14	118.0 (2)
C8	C13	C11	122.13 (18)
N2	C16	C10	178.5 (2)
C4	C3	C2	117.74 (19)
N1	C15	C9	178.2 (2)
C2	C1	C11	119.30 (19)
C11	C12	C9	118.6 (2)
C4	C12	C11	120.36 (18)
C4	C12	C9	121.0 (2)
F2	C7	C8	117.84 (18)
F2	C7	C6	117.8 (2)
C8	C7	C6	124.3 (2)
C6	C5	C14	120.8 (2)
C14	C10	C16	118.6 (2)
C9	C10	C14	121.6 (2)
C9	C10	C16	119.7 (2)

**Table 6:** Torsion angles for 3,6-difluorophenanthrene-9,10-dicarbonitrile.

A	B	C	D	Angle/˚
F1	C2	C3	C4	−178.63 (18)
F1	C2	C1	C11	178.91 (17)
C13	C14	C5	C6	−0.7 (3)
C13	C14	C10	C9	−0.2 (3)
C13	C14	C10	C16	178.45 (17)
C13	C8	C7	F2	179.88 (18)
C13	C8	C7	C6	−0.8 (3)
C13	C11	C1	C2	179.90 (18)
C13	C11	C12	C4	−179.49 (18)
C13	C11	C12	C9	0.1 (3)
C3	C2	C1	C11	−0.4 (3)
C3	C4	C12	C11	−0.4 (3)
C3	C4	C12	C9	179.99 (18)
C15	C9	C12	C11	−177.39 (18)
C15	C9	C12	C4	2.2 (3)
C15	C9	C10	C14	177.43 (18)
C15	C9	C10	C16	−1.2 (3)
C1	C2	C3	C4	0.7 (3)
C1	C11	C13	C14	178.46 (18)
C1	C11	C13	C8	−1.4 (3)
C1	C11	C12	C4	0.7 (3)
C1	C11	C12	C9	−179.70 (17)
C12	C11	C13	C14	−1.3 (3)
C12	C11	C13	C8	178.78 (17)
C12	C11	C1	C2	−0.3 (3)
C12	C4	C3	C2	−0.3 (3)
C12	C9	C10	C14	−1.1 (3)
C12	C9	C10	C16	−179.69 (18)
C7	C8	C13	C14	0.4 (3)
C7	C8	C13	C11	−179.76 (18)
C7	C6	C5	C14	0.3 (3)
C5	C14	C13	C8	0.4 (3)
C5	C14	C13	C11	−179.5 (2)
C5	C14	C10	C9	−179.30 (19)
C5	C14	C10	C16	−0.7 (3)
C5	C6	C7	F2	179.81 (19)
C5	C6	C7	C8	0.5 (3)
C10	C14	C13	C8	−178.74 (19)
C10	C14	C13	C11	1.4 (3)
C10	C14	C5	C6	178.40 (19)
C10	C9	C12	C11	1.1 (3)
C10	C9	C12	C4	−179.31 (19)

**Table 7:** Bond lengths for 3,6-dimethylphenanthrene-9,10-dicarbonitrile.

Atom	Atom	Length/Å
N1	C15	1.1529 (18)
N2	C16	1.151 (2)
N1A	C0AA	1.1496 (19)
N2A	C16A	1.1516 (19)
C14	C13	1.4138 (17)
C14	C10	1.4358 (16)
C14	C5	1.4125 (17)
C13	C11	1.4603 (16)
C13	C8	1.4089 (16)
C14A	C13A	1.4233 (17)
C14A	C10A	1.4317 (17)
C14A	C5A	1.4154 (17)
C10	C9	1.3776 (17)
C10	C16	1.4387 (18)
C12A	C11A	1.4147 (17)
C12A	C9A	1.4360 (17)
C12A	C4A	1.4148 (17)
C13A	C11A	1.4579 (16)
C13A	C8A	1.4059 (17)
C11	C12	1.4156 (16)
C11	C1	1.4093 (17)
C11A	C1A	1.4142 (17)
C9A	C10A	1.3741 (17)
C9A	C0AA	1.4366 (17)
C12	C9	1.4344 (17)
C12	C4	1.4150 (17)
C4A	C3A	1.3732 (19)
C2	C1	1.3836 (18)
C2	C17	1.5063 (16)
C2	C3	1.4163 (18)
C3A	C2A	1.4095 (18)
C10A	C16A	1.4426 (17)
C6A	C7A	1.4129 (18)
C6A	C5A	1.3654 (18)
C7A	C8A	1.3799 (17)
C7A	C15A	1.5041 (17)
C15	C9	1.4393 (17)
C1A	C2A	1.3842 (17)
C2A	C18A	1.5034 (17)
C7	C8	1.3818 (17)
C7	C6	1.4104 (18)
C7	C18	1.5018 (17)
C5	C6	1.3723 (18)
C4	C3	1.3647 (19)

**Table 8:** Bond angles for 3,6-dimethylphenanthrene-9,10-dicarbonitrile.

Atom	Atom	Atom	Angle/˚
C13	C14	C10	119.12 (11)
C5	C14	C13	120.07 (11)
C5	C14	C10	120.79 (11)
C14	C13	C11	119.92 (11)
C8	C13	C14	117.99 (11)
C8	C13	C11	122.08 (11)
C13A	C14A	C10A	118.98 (11)
C5A	C14A	C13A	119.46 (11)
C5A	C14A	C10A	121.55 (11)
C14	C10	C16	119.19 (11)
C9	C10	C14	121.00 (12)
C9	C10	C16	119.80 (11)
C11A	C12A	C9A	118.85 (11)
C11A	C12A	C4A	119.93 (11)
C4A	C12A	C9A	121.20 (11)
C14A	C13A	C11A	119.57 (11)
C8A	C13A	C14A	117.83 (11)
C8A	C13A	C11A	122.59 (11)
C12	C11	C13	119.43 (11)
C1	C11	C13	122.35 (11)
C1	C11	C12	118.22 (11)
C12A	C11A	C13A	119.87 (11)
C1A	C11A	C12A	117.91 (11)
C1A	C11A	C13A	122.22 (11)
C12A	C9A	C0AA	119.83 (11)
C10A	C9A	C12A	121.45 (11)
C10A	C9A	C0AA	118.72 (11)
C11	C12	C9	119.24 (11)
C4	C12	C11	119.37 (12)
C4	C12	C9	121.39 (11)
C3A	C4A	C12A	120.36 (12)
C1	C2	C17	121.30 (12)
C1	C2	C3	118.42 (12)
C3	C2	C17	120.26 (12)
C4A	C3A	C2A	120.87 (12)
C14A	C10A	C16A	119.85 (11)
C9A	C10A	C14A	121.18 (11)
C9A	C10A	C16A	118.95 (11)
C2	C1	C11	122.22 (12)
C5A	C6A	C7A	120.60 (12)
C6A	C7A	C15A	120.51 (12)
C8A	C7A	C6A	118.94 (12)
C8A	C7A	C15A	120.54 (12)
N1A	C0AA	C9A	178.19 (15)
N1	C15	C9	178.78 (15)
C2A	C1A	C11A	122.04 (12)
C6A	C5A	C14A	120.83 (12)
C3A	C2A	C18A	120.06 (12)
C1A	C2A	C3A	118.87 (12)
C1A	C2A	C18A	121.06 (12)
C10	C9	C12	121.24 (11)
C10	C9	C15	118.61 (11)
C12	C9	C15	120.14 (11)
C7A	C8A	C13A	122.34 (12)
N2	C16	C10	178.82 (14)
C8	C7	C6	119.16 (11)
C8	C7	C18	120.74 (12)
C6	C7	C18	120.11 (11)
C6	C5	C14	120.27 (12)
N2A	C16A	C10A	177.61 (15)
C7	C8	C13	121.87 (12)
C3	C4	C12	120.84 (12)
C5	C6	C7	120.63 (11)
C4	C3	C2	120.90 (11)

**Table 9:** Torsion angles for 3,6-dimethylphenanthrene-9,10-dicarbonitrile.

A	B	C	D	Angle/˚
C14	C13	C11	C12	1.27 (17)
C14	C13	C11	C1	−179.03 (11)
C14	C13	C8	C7	0.90 (18)
C14	C10	C9	C12	1.66 (19)
C14	C10	C9	C15	−176.83 (11)
C14	C5	C6	C7	0.75 (19)
C13	C14	C10	C9	0.34 (18)
C13	C14	C10	C16	−178.50 (11)
C13	C14	C5	C6	0.16 (18)
C13	C11	C12	C9	0.68 (17)
C13	C11	C12	C4	−179.05 (11)
C13	C11	C1	C2	179.80 (11)
C14A	C13A	C11A	C12A	−2.31 (18)
C14A	C13A	C11A	C1A	177.20 (11)
C14A	C13A	C8A	C7A	0.71 (19)
C10	C14	C13	C11	−1.79 (17)
C10	C14	C13	C8	177.72 (11)
C10	C14	C5	C6	−178.51 (12)
C12A	C11A	C1A	C2A	0.96 (19)
C12A	C9A	C10A	C14A	−3.33 (19)
C12A	C9A	C10A	C16A	175.19 (11)
C12A	C4A	C3A	C2A	0.5 (2)
C13A	C14A	C10A	C9A	1.94 (18)
C13A	C14A	C10A	C16A	−176.56 (11)
C13A	C14A	C5A	C6A	0.31 (19)
C13A	C11A	C1A	C2A	−178.57 (12)
C11	C13	C8	C7	−179.60 (11)
C11	C12	C9	C10	−2.16 (18)
C11	C12	C9	C15	176.31 (11)
C11	C12	C4	C3	−0.75 (19)
C11A	C12A	C9A	C10A	1.81 (19)
C11A	C12A	C9A	C0AA	−177.59 (11)
C11A	C12A	C4A	C3A	−1.02 (19)
C11A	C13A	C8A	C7A	179.82 (12)
C11A	C1A	C2A	C3A	−1.46 (19)
C11A	C1A	C2A	C18A	177.27 (12)
C9A	C12A	C11A	C13A	1.02 (18)
C9A	C12A	C11A	C1A	−178.52 (11)
C9A	C12A	C4A	C3A	177.76 (12)
C12	C11	C1	C2	−0.50 (18)
C12	C4	C3	C2	−0.5 (2)
C4A	C12A	C11A	C13A	179.83 (11)
C4A	C12A	C11A	C1A	0.29 (18)
C4A	C12A	C9A	C10A	−176.99 (12)
C4A	C12A	C9A	C0AA	3.61 (19)
C4A	C3A	C2A	C1A	0.7 (2)
C4A	C3A	C2A	C18A	−178.04 (12)
C10A	C14A	C13A	C11A	0.87 (17)
C10A	C14A	C13A	C8A	180.00 (11)
C10A	C14A	C5A	C6A	179.49 (12)
C1	C11	C12	C9	−179.03 (11)
C1	C11	C12	C4	1.24 (17)
C1	C2	C3	C4	1.3 (2)
C6A	C7A	C8A	C13A	−0.11 (19)
C7A	C6A	C5A	C14A	0.3 (2)
C0AA	C9A	C10A	C14A	176.07 (11)
C0AA	C9A	C10A	C16A	−5.41 (18)
C5A	C14A	C13A	C11A	−179.93 (11)
C5A	C14A	C13A	C8A	−0.80 (18)
C5A	C14A	C10A	C9A	−177.24 (12)
C5A	C14A	C10A	C16A	4.26 (18)
C5A	C6A	C7A	C8A	−0.4 (2)
C5A	C6A	C7A	C15A	−179.92 (13)
C9	C12	C4	C3	179.53 (12)
C8A	C13A	C11A	C12A	178.60 (11)
C8A	C13A	C11A	C1A	−1.88 (19)
C16	C10	C9	C12	−179.51 (11)
C16	C10	C9	C15	2.00 (18)
C5	C14	C13	C11	179.53 (11)
C5	C14	C13	C8	−0.96 (17)
C5	C14	C10	C9	179.02 (11)
C5	C14	C10	C16	0.18 (18)
C8	C13	C11	C12	−178.22 (11)
C8	C13	C11	C1	1.48 (18)
C8	C7	C6	C5	−0.82 (19)
C4	C12	C9	C10	177.57 (12)
C4	C12	C9	C15	−3.97 (19)
C17	C2	C1	C11	178.18 (12)
C17	C2	C3	C4	−177.67 (12)
C6	C7	C8	C13	−0.03 (19)
C3	C2	C1	C11	−0.73 (19)
C15A	C7A	C8A	C13A	179.40 (12)
C18	C7	C8	C13	179.98 (12)
C18	C7	C6	C5	179.17 (12)

**Table 10:** Bond lengths for 3,6-dimethoxyphenanthrene-9,10-dicarbonitrile.

Atom	Atom	Length/Å
C1	C2	1.378 (2)
C1	C11	1.408 (2)
C2	C3	1.408 (2)
C2	O1	1.3624 (19)
C3	C4	1.361 (2)
C4	C12	1.418 (2)
C5	C6	1.372 (2)
C5	C14	1.411 (2)
C6	C7	1.402 (2)
C7	C8	1.384 (2)
C7	O2	1.3643 (19)
C8	C13	1.404 (2)
C9	C10	1.373 (2)
C9	C12	1.433 (2)
C9	C15	1.440 (2)
C10	C14	1.435 (2)
C10	C16	1.439 (2)
C11	C12	1.414 (2)
C11	C13	1.461 (2)
C13	C14	1.418 (2)
C15	N1	1.145 (2)
C16	N2	1.145 (2)
C17	O1	1.4361 (19)
C18	O2	1.431 (2)

**Table 11:** Bond angles for 3,6-dimethoxyphenanthrene-9,10-dicarbonitrile.

Atom	Atom	Atom	Angle/˚
C2	C1	C11	120.24 (15)
C1	C2	C3	120.88 (15)
O1	C2	C1	124.35 (15)
O1	C2	C3	114.77 (14)
C4	C3	C2	119.73 (15)
C3	C4	C12	120.82 (15)
C6	C5	C14	121.23 (16)
C5	C6	C7	119.45 (15)
C8	C7	C6	120.53 (15)
O2	C7	C6	124.23 (15)
O2	C7	C8	115.24 (14)
C7	C8	C13	120.95 (15)
C10	C9	C12	121.44 (15)
C10	C9	C15	119.59 (15)
C12	C9	C15	118.95 (14)
C9	C10	C14	120.93 (15)
C9	C10	C16	119.10 (15)
C14	C10	C16	119.95 (15)
C1	C11	C12	118.90 (14)
C1	C11	C13	121.80 (15)
C12	C11	C13	119.31 (14)
C4	C12	C9	121.21 (15)
C11	C12	C4	119.43 (15)
C11	C12	C9	119.36 (14)
C8	C13	C11	121.68 (15)
C8	C13	C14	118.50 (15)
C14	C13	C11	119.82 (15)
C5	C14	C10	121.53 (15)
C5	C14	C13	119.35 (15)
C13	C14	C10	119.12 (15)
N1	C15	C9	179.7 (2)
N2	C16	C10	178.49 (19)
C2	O1	C17	116.72 (12)
C7	O2	C18	117.18 (13)

**Table 12:** Torsion angles for 3,6-dimethoxyphenanthrene-9,10-dicarbonitrile.

A	B	C	D	Angle/˚
C1	C2	C3	C4	0.4 (2)
C1	C2	O1	C17	−0.9 (2)
C1	C11	C12	C4	0.6 (2)
C1	C11	C12	C9	−179.17 (14)
C1	C11	C13	C8	−1.3 (2)
C1	C11	C13	C14	179.34 (15)
C2	C1	C11	C12	−0.5 (2)
C2	C1	C11	C13	179.13 (15)
C2	C3	C4	C12	−0.2 (2)
C3	C2	O1	C17	178.81 (14)
C3	C4	C12	C9	179.49 (15)
C3	C4	C12	C11	−0.3 (2)
C5	C6	C7	C8	0.3 (3)
C5	C6	C7	O2	−179.30 (16)
C6	C5	C14	C10	179.62 (16)
C6	C5	C14	C13	−0.2 (3)
C6	C7	C8	C13	−0.4 (2)
C6	C7	O2	C18	2.7 (2)
C7	C8	C13	C11	−179.24 (15)
C7	C8	C13	C14	0.1 (2)
C8	C7	O2	C18	−176.96 (14)
C8	C13	C14	C5	0.1 (2)
C8	C13	C14	C10	−179.66 (15)
C9	C10	C14	C5	−178.29 (16)
C9	C10	C14	C13	1.5 (2)
C10	C9	C12	C4	−179.82 (16)
C10	C9	C12	C11	0.0 (2)
C11	C1	C2	C3	0.0 (2)
C11	C1	C2	O1	179.62 (15)
C11	C13	C14	C5	179.52 (15)
C11	C13	C14	C10	−0.3 (2)
C12	C9	C10	C14	−1.4 (2)
C12	C9	C10	C16	176.95 (15)
C12	C11	C13	C8	178.30 (15)
C12	C11	C13	C14	−1.1 (2)
C13	C11	C12	C4	−178.97 (15)
C13	C11	C12	C9	1.2 (2)
C14	C5	C6	C7	−0.1 (3)
C15	C9	C10	C14	−179.70 (15)
C15	C9	C10	C16	−1.4 (2)
C15	C9	C12	C4	−1.5 (2)
C15	C9	C12	C11	178.32 (15)
C16	C10	C14	C5	3.4 (2)
C16	C10	C14	C13	−176.81 (15)
O1	C2	C3	C4	−179.32 (14)
O2	C7	C8	C13	179.30 (14)

**Table 13:** Intermolecular distances for 3,6-difluorophenanthrene-9,10-dicarbonitrile.

Atom	Atom	Length/Å
F1	H5#1	2.493 (2)
H1	N2#1	2.577 (2)
H8	N2#1	2.628 (2)
F2	H4#2	2.586 (2)
H6	N1#2	2.658 (2)
H5	F1#3	2.493 (2)
N2	H1#3	2.577 (2)
N2	H8#3	2.628 (2)
N1	H6#4	2.658 (2)
H4	F2#4	2.586 (2)

**Table 14:** Intermolecular distances for 3,6-dimethylphenanthrene-9,10-dicarbonitrile.

Atom	Atom	Length/Å
N1	H3A#1	2.728 (2)
N1	H18B#2	2.943 (2)
H8	N1A#3	2.545 (2)
H18B	N1#4	2.943 (2)
N1A	H8#5	2.545 (2)
N2A	H18E#6	2.619 (2)
H18E	N2A#7	2.619 (2)
H3A	N1#8	2.728 (2)

**Table 15:** Intermolecular distances for 3,6-dimethoxyphenanthrene-9,10-dicarbonitrile.

Atom	Atom	Length/Å
N1	H1#1	2.637 (2)
N1	H8#1	2.450 (2)
N2	H17b#1	2.591 (2)
H17b	N2#2	2.591 (2)
H1	N1#2	2.637 (2)
H8	N1#2	2.450 (2)

## 2 Experimental design, materials, and methods

Phenanthrene-9,10-dicarbonitriles were obtained by the previously published procedures [[1], [2], [3], [4], [5]]. The crystals of appropriate quality were obtained at room temperature from ethanol solution. The X-ray diffraction data were collected on an Agilent Technologies Excalibur Eos and Supernova Atlas diffractometers. The temperature for all experiments was kept at 100 K. The structures have been solved by the direct methods and refined by means of the SHELXL–97 [6] program incorporated in the OLEX^2^ program package [7]. The carbon-bound H atoms were placed in calculated positions and were included in the refinement in the ‘riding’ model approximation, with U_iso_(H) set to 1.5U_eq_(C) and C–H 0.96 Å for CH_3_ groups, U_iso_(H) set to 1.2U_eq_(C) and C–H 0.93 Å for the CH groups, and U_iso_(H) set to 1.2U_eq_(N) and N–H 0.86 Å for the NH groups. Empirical absorption correction was applied in CrysAlisPro program complex [8] using spherical harmonics, implemented in SCALE3 ABSPACK scaling algorithm.
