# Supplementary material for: Structural data of phenanthrene-9,10-dicarbonitriles
Source: Data Brief. 2019 Oct 5;27:104605. doi: 10.1016/j.dib.2019.104605 (PMC6812052; doi:10.1016/j.dib.2019.104605)
Supplement: Multimedia component 1 [file mmc1.zip › checkCIF_6_ССDC 1821026.pdf]

checkCIF (basic structural check) running

Checking for embedded fcf data in CIF ...
Found embedded fcf data in CIF. Extracting fcf data from uploaded CIF, please wait . . .

checkCIF/PLATON (basic structural check)

Structure factors have been supplied for datablock(s) tgc24

THIS REPORT IS FOR GUIDANCE ONLY. IF USED AS PART OF A REVIEW PROCEDURE FOR PUBLICATION, IT SHOULD NOT REPLACE THE EXPERTISE OF AN EXPERIENCED CRYSTALLOGRAPHIC REFEREE.

No syntax errors found. CIF dictionary
Please wait while processing .... Interpreting this report
Structure factor report

Datablock: tgc24

```
Bond precision:      C-C = 0.0019 A              Wavelength=1.54184
Cell:          a=11.1090(2)    b=17.7567(3)    c=14.0781(3)
               alpha=90        beta=109.077(2) gamma=90
Temperature:100 K
```

	Calculated	Reported
Volume	2624.52(9)	2624.52(9)
Space group	P 21/c	P 1 21/c 1
Hall group	-P 2ybc	-P 2ybc
Moiety formula	C18 H12 N2	C18 H12 N2
Sum formula	C18 H12 N2	C18 H12 N2
Mr	256.30	256.30
Dx,g cm-3	1.297	1.297
Z	8	8
Mu (mm-1)	0.601	0.601
F000	1072.0	1072.0
F000'	1074.90	
h,k,lmax	13,21,17	13,21,17
Nref	5204	5113
Tmin,Tmax	0.891,0.953	0.653,1.000
Tmin'	0.876	

```
Correction method= # Reported T Limits: Tmin=0.653
Tmax=1.000 AbsCorr = MULTI-SCAN
Data completeness= 0.983        Theta(max)= 72.482
R(reflections)= 0.0456( 4300)      wR2(reflections)= 0.1323( 5113)
S = 1.034              Npar= 366
```

The following ALERTS were generated. Each ALERT has the format
test-name_ALERT_alert-type_alert-level.

Click on the hyperlinks for more details of the test.

Alert level C

PLAT250_ALERT_2_C Large U3/U1 Ratio for Average U(i,j) Tensor .... 2.2 Note
PLAT906_ALERT_3_C Large K Value in the Analysis of Variance ...... 4.433 Check
PLAT911_ALERT_3_C Missing FCF Refl Between Thmin & STh/L= 0.600 53 Report

Alert level G

PLAT230_ALERT_2_G Hirshfeld Test Diff for C0AA --C9A . 5.3 s.u.
PLAT230_ALERT_2_G Hirshfeld Test Diff for C10A --C16A . 5.6 s.u.
PLAT333_ALERT_2_G Large Aver C6-Ring C-C Dist. C9 -C12 1.42 Ang.
PLAT333_ALERT_2_G Large Aver C6-Ring C-C Dist. C9A -C12A 1.42 Ang.
PLAT720_ALERT_4_G Number of Unusual/Non-Standard Labels .... 1 Note
PLAT912_ALERT_4_G Missing # of FCF Reflections Above STh/L= 0.600 38 Note
PLAT933_ALERT_2_G Number of OMIT Records in Embedded .res File ... 9 Note
PLAT978_ALERT_2_G Number C-C Bonds with Positive Residual Density. 8 Info

0 **ALERT level A** = Most likely a serious problem - resolve or explain
0 **ALERT level B** = A potentially serious problem, consider carefully
3 **ALERT level C** = Check. Ensure it is not caused by an omission or oversight
8 **ALERT level G** = General information/check it is not something unexpected

0 ALERT type 1 CIF construction/syntax error, inconsistent or missing data
7 ALERT type 2 Indicator that the structure model may be wrong or deficient
2 ALERT type 3 Indicator that the structure quality may be low
2 ALERT type 4 Improvement, methodology, query or suggestion
0 ALERT type 5 Informative message, check

Validation response form

Please find below a validation response form (VRF) that can be filled in and pasted into your CIF.

```
# start Validation Reply Form
_vrf_PLAT250_tgc24
;
PROBLEM: Large U3/U1 Ratio for Average U(i,j) Tensor ....       2.2 Note
RESPONSE: ...
;
_vrf_PLAT906_tgc24
;
PROBLEM: Large K Value in the Analysis of Variance ......      4.433 Check
RESPONSE: ...
;
_vrf_PLAT911_tgc24
;
PROBLEM: Missing FCF Refl Between Thmin & STh/L=  0.600        53 Report
RESPONSE: ...
;
# end Validation Reply Form
```

It is advisable to attempt to resolve as many as possible of the alerts in all categories. Often the minor alerts point to easily fixed oversights, errors and omissions in your CIF or refinement strategy, so attention to these fine details can be worthwhile. In order to resolve some of the more serious problems it may be necessary to carry out additional measurements or structure refinements. However, the purpose of your study may justify the reported deviations and the more serious of these should normally be commented upon in the discussion or experimental section of a paper or in the "special_details" fields of the CIF. checkCIF was carefully designed to identify outliers and unusual parameters, but every test has its limitations and alerts that are not important in a particular case may appear. Conversely, the absence of alerts does not guarantee there are no aspects of the results needing attention. It is up to the individual to

critically assess their own results and, if necessary, seek expert advice.

Publication of your CIF in IUCr journals

A basic structural check has been run on your CIF. These basic checks will be run on all CIFs submitted for publication in IUCr journals (*Acta Crystallographica*, *Journal of Applied Crystallography*, *Journal of Synchrotron Radiation*); however, if you intend to submit to *Acta Crystallographica Section C* or *E* or *IUCrData*, you should make sure that full publication checks are run on the final version of your CIF prior to submission.

Publication of your CIF in other journals

Please refer to the *Notes for Authors* of the relevant journal for any special instructions relating to CIF submission.

PLATON version of 13/08/2017; check.def file version of 12/12/2017

Datablock tgc24 - ellipsoid plot

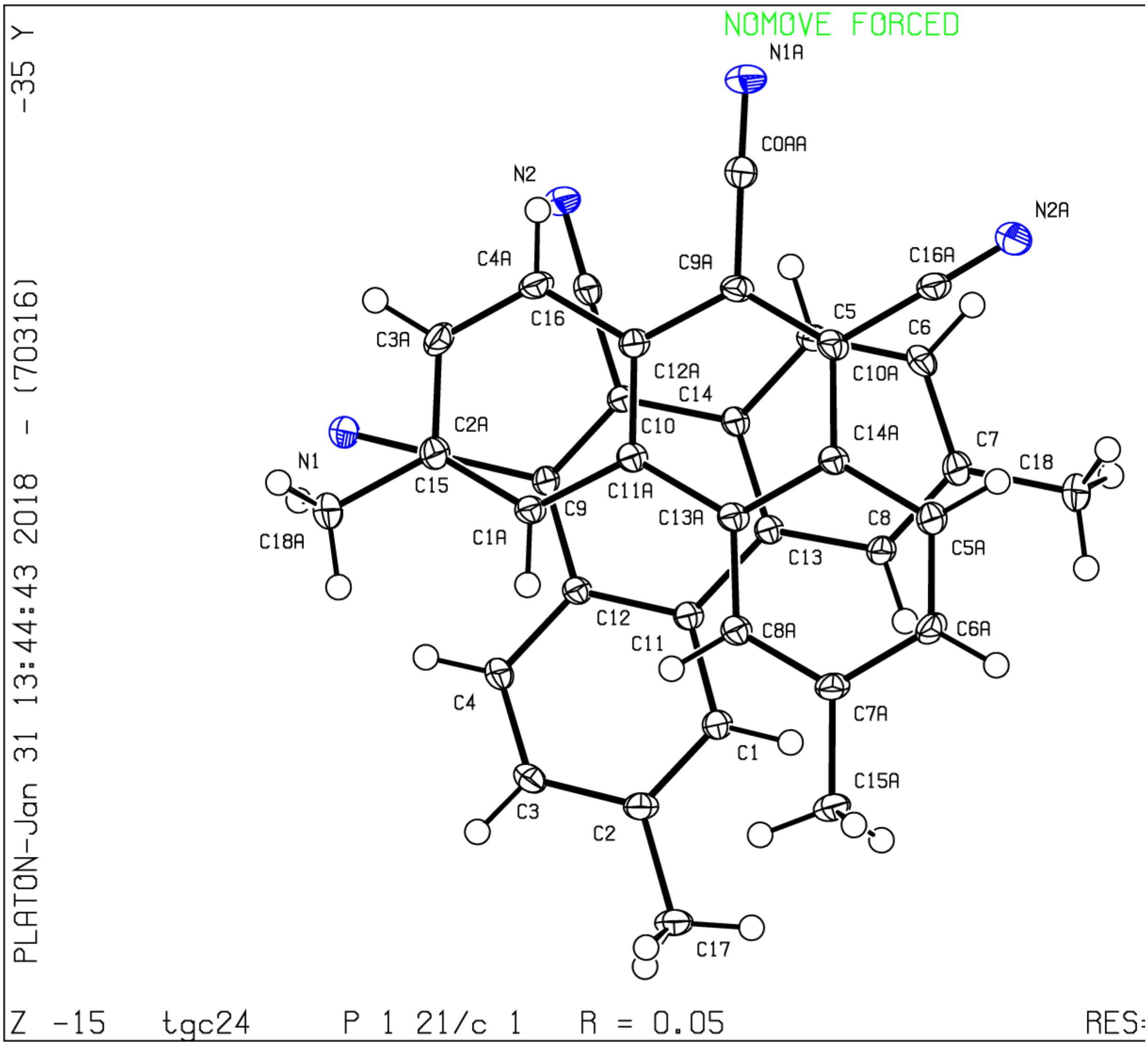

Download CIF editor (publCIF) from the IUCr
Download CIF editor (enCIFer) from the CCDC
Test a new CIF entry
