# Supplementary material for: Structural data of phenanthrene-9,10-dicarbonitriles
Source: Data Brief. 2019 Oct 5;27:104605. doi: 10.1016/j.dib.2019.104605 (PMC6812052; doi:10.1016/j.dib.2019.104605)
Supplement: Multimedia component 1 [file mmc1.zip › checkcif_2_ССDC 1821025.pdf]

checkCIF (basic structural check) running

Checking for embedded fcf data in CIF ...
Found embedded fcf data in CIF. Extracting fcf data from uploaded CIF, please wait . .

checkCIF/PLATON (basic structural check)

Structure factors have been supplied for datablock(s) tgc22

THIS REPORT IS FOR GUIDANCE ONLY. IF USED AS PART OF A REVIEW PROCEDURE FOR PUBLICATION, IT SHOULD NOT REPLACE THE EXPERTISE OF AN EXPERIENCED CRYSTALLOGRAPHIC REFEREE.

No syntax errors found. CIF dictionary
Please wait while processing .... Interpreting this report
Structure factor report

Datablock: tgc22

```
Bond precision:      C-C = 0.0030 A            Wavelength=1.54184
Cell:        a=3.73677(9)   b=20.2474(5)   c=15.6017(4)
             alpha=90       beta=94.366(2) gamma=90
Temperature: 100 K
```

	Calculated	Reported
Volume	1177.00(5)	1177.00(5)
Space group	C c	C 1 c 1
Hall group	C -2yc	C -2yc
Moiety formula	C16 H6 F2 N2	C16 H6 F2 N2
Sum formula	C16 H6 F2 N2	C16 H6 F2 N2
Mr	264.23	264.23
Dx,g cm-3	1.491	1.491
Z	4	4
Mu (mm-1)	0.946	0.946
F000	536.0	536.0
F000'	537.89	
h,k,lmax	4,23,18	4,23,18
Nref	2007[1009]	1593
Tmin,Tmax	0.853,0.927	0.839,1.000
Tmin'	0.812	

```
Correction method= # Reported T Limits: Tmin=0.839
Tmax=1.000 AbsCorr = MULTI-SCAN
Data completeness= 1.58/0.79   Theta(max)= 64.956
R(reflections)= 0.0298( 1562)      wR2(reflections)= 0.0752( 1593)
S = 1.070              Npar= 181
```

The following ALERTS were generated. Each ALERT has the format
test-name_ALERT_alert-type_alert-level.

Click on the hyperlinks for more details of the test.

Alert level C

THETM01_ALERT_3_C The value of sine(theta_max)/wavelength is less than 0.590
Calculated sin(theta_max)/wavelength = 0.5876
PLAT089_ALERT_3_C Poor Data / Parameter Ratio (Zmax < 18) .... 5.57 Note
PLAT915_ALERT_3_C No Flack x Check Done: Low Friedel Pair Coverage 59 %

Alert level G

PLAT333_ALERT_2_G Large Aver C6-Ring C-C Dist. C9 -C12 1.42 Ang.
PLAT909_ALERT_3_G Percentage of I>2sig(I) Data at Theta(Max) Still 97% Note
PLAT978_ALERT_2_G Number C-C Bonds with Positive Residual Density. 4 Info

0 **ALERT level A** = Most likely a serious problem - resolve or explain
0 **ALERT level B** = A potentially serious problem, consider carefully
3 **ALERT level C** = Check. Ensure it is not caused by an omission or oversight
3 **ALERT level G** = General information/check it is not something unexpected

0 ALERT type 1 CIF construction/syntax error, inconsistent or missing data
2 ALERT type 2 Indicator that the structure model may be wrong or deficient
4 ALERT type 3 Indicator that the structure quality may be low
0 ALERT type 4 Improvement, methodology, query or suggestion
0 ALERT type 5 Informative message, check

Validation response form

Please find below a validation response form (VRF) that can be filled in and pasted into your CIF.

```
# start Validation Reply Form
_vrf_THETM01_tgc22
;
PROBLEM: The value of sine(theta_max)/wavelength is less than 0.590
RESPONSE: ...
;
_vrf_PLAT089_tgc22
;
PROBLEM: Poor Data / Parameter Ratio (Zmax < 18) ........       5.57 Note
RESPONSE: ...
;
_vrf_PLAT915_tgc22
;
PROBLEM: No Flack x Check Done: Low Friedel Pair Coverage         59 %
RESPONSE: ...
;
# end Validation Reply Form
```

It is advisable to attempt to resolve as many as possible of the alerts in all categories. Often the minor alerts point to easily fixed oversights, errors and omissions in your CIF or refinement strategy, so attention to these fine details can be worthwhile. In order to resolve some of the more serious problems it may be necessary to carry out additional measurements or structure refinements. However, the purpose of your study may justify the reported deviations and the more serious of these should normally be commented upon in the discussion or experimental section of a paper or in the "special_details" fields of the CIF. checkCIF was carefully designed to identify outliers and unusual parameters, but every test has its limitations and alerts that are not important in a particular case may appear. Conversely, the absence of alerts does not guarantee there are no aspects of the results needing attention. It is up to the individual to critically assess their own results and, if necessary, seek expert advice.

Publication of your CIF in IUCr journals

A basic structural check has been run on your CIF. These basic checks will be run on all CIFs submitted for publication in IUCr journals (*Acta Crystallographica*, *Journal of Applied Crystallography*, *Journal of Synchrotron Radiation*); however, if you intend to submit to *Acta Crystallographica Section C* or *E* or *IUCrData*, you should make sure that full publication checks are run on the final version of your CIF prior to submission.

Publication of your CIF in other journals

Please refer to the *Notes for Authors* of the relevant journal for any special instructions relating to CIF submission.

PLATON version of 13/08/2017; check.def file version of 12/12/2017

Datablock tgc22 - ellipsoid plot

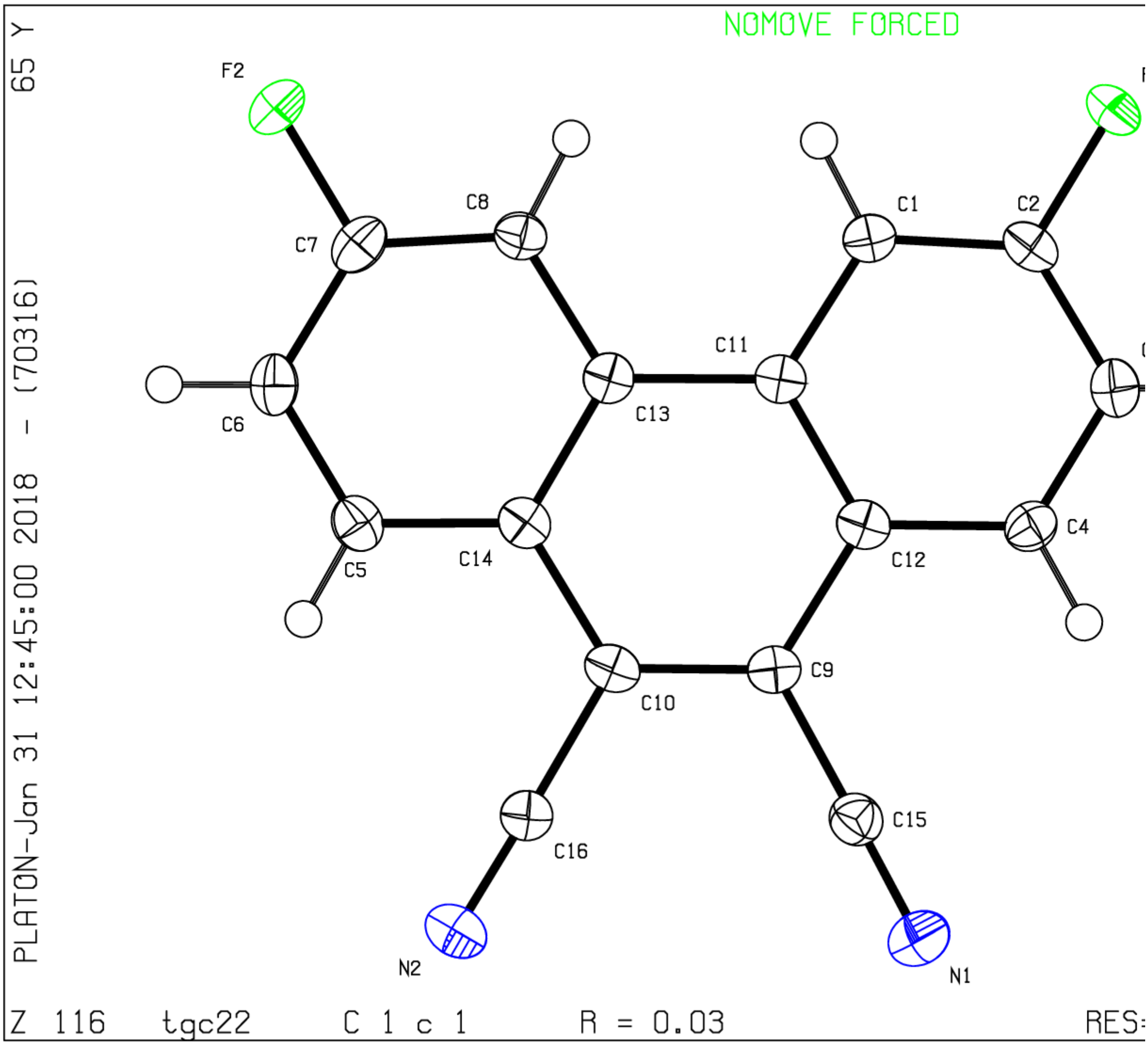

Download CIF editor (publCIF) from the IUCr
Download CIF editor (enCIFer) from the CCDC
Test a new CIF entry
