# Supplementary material for: Structural data of phenanthrene-9,10-dicarbonitriles
Source: Data Brief. 2019 Oct 5;27:104605. doi: 10.1016/j.dib.2019.104605 (PMC6812052; doi:10.1016/j.dib.2019.104605)

checkCIF/PLATON report

Structure factors have been supplied for datablock(s) tgc_20

THIS REPORT IS FOR GUIDANCE ONLY. IF USED AS PART OF A REVIEW PROCEDURE FOR PUBLICATION, IT SHOULD NOT REPLACE THE EXPERTISE OF AN EXPERIENCED CRYSTALLOGRAPHIC REFEREE.

No syntax errors found.   CIF dictionary   Interpreting this report

Datablock: tgc_20

```
Bond precision:  C-C = 0.0020 A          Wavelength=0.71073

Cell:            a=7.7888(4)      b=21.9465(10)       c=7.8773(4)
                 alpha=90         beta=98.517(4)      gamma=90
Temperature:     100 K

                Calculated                 Reported
Volume          1331.67(11)                1331.66(11)
Space group     P 21/c                     P 1 21/c 1
Hall group      -P 2ybc                    -P 2ybc
Moiety formula  C18 H12 N2 O2              C18 H12 N2 O2
Sum formula     C18 H12 N2 O2              C18 H12 N2 O2
Mr              288.30                     288.30
Dx,g cm-3       1.438                      1.438
Z               4                          4
Mu (mm-1)       0.096                      0.096
F000            600.0                      600.0
F000'           600.26
h,k,lmax        9,28,10                    9,28,9
Nref            2891                       2769
Tmin,Tmax       0.979,0.989                0.990,1.000
Tmin'           0.977

Correction method= # Reported T Limits: Tmin=0.990 Tmax=1.000
AbsCorr = MULTI-SCAN

Data completeness= 0.958          Theta(max)= 26.999

R(reflections)= 0.0451( 2159)     wR2(reflections)= 0.1109( 2769)

S = 1.033                  Npar= 201
```

The following ALERTS were generated. Each ALERT has the format
test-name_ALERT_alert-type_alert-level.
Click on the hyperlinks for more details of the test.

Alert level C

```
CRYSC01_ALERT_1_C No recognised colour has been given for crystal colour.
PLAT029_ALERT_3_C _diffrn_measured_fraction_theta_full value Low .      0.967 Why?
PLAT906_ALERT_3_C Large K Value in the Analysis of Variance ......      3.770 Check
PLAT911_ALERT_3_C Missing FCF Refl Between Thmin & STh/L=  0.600         70 Report
```

Alert level G

```
PLAT333_ALERT_2_G Large Aver C6-Ring C-C Dist. C9     -C12           1.42 Ang.
PLAT910_ALERT_3_G Missing # of FCF Reflection(s) Below Theta(Min).          1 Note
PLAT912_ALERT_4_G Missing # of FCF Reflections Above STh/L=  0.600         45 Note
PLAT978_ALERT_2_G Number C-C Bonds with Positive Residual Density.         17 Info
```

```
   0 ALERT level A = Most likely a serious problem - resolve or explain
   0 ALERT level B = A potentially serious problem, consider carefully
   4 ALERT level C = Check. Ensure it is not caused by an omission or oversight
   4 ALERT level G = General information/check it is not something unexpected

   1 ALERT type 1 CIF construction/syntax error, inconsistent or missing data
   2 ALERT type 2 Indicator that the structure model may be wrong or deficient
   4 ALERT type 3 Indicator that the structure quality may be low
   1 ALERT type 4 Improvement, methodology, query or suggestion
   0 ALERT type 5 Informative message, check
```

It is advisable to attempt to resolve as many as possible of the alerts in all categories. Often the minor alerts point to easily fixed oversights, errors and omissions in your CIF or refinement strategy, so attention to these fine details can be worthwhile. In order to resolve some of the more serious problems it may be necessary to carry out additional measurements or structure refinements. However, the purpose of your study may justify the reported deviations and the more serious of these should normally be commented upon in the discussion or experimental section of a paper or in the "special_details" fields of the CIF. checkCIF was carefully designed to identify outliers and unusual parameters, but every test has its limitations and alerts that are not important in a particular case may appear. Conversely, the absence of alerts does not guarantee there are no aspects of the results needing attention. It is up to the individual to critically assess their own results and, if necessary, seek expert advice.

Publication of your CIF in IUCr journals

A basic structural check has been run on your CIF. These basic checks will be run on all CIFs submitted for publication in IUCr journals (*Acta Crystallographica*, *Journal of Applied Crystallography*, *Journal of Synchrotron Radiation*); however, if you intend to submit to *Acta Crystallographica Section C* or *E* or *IUCrData*, you should make sure that full publication checks are run on the final version of your CIF prior to submission.

Publication of your CIF in other journals

Please refer to the *Notes for Authors* of the relevant journal for any special instructions relating to CIF submission.

PLATON version of 13/08/2017; check.def file version of 12/12/2017

Datablock tgc_20 - ellipsoid plot

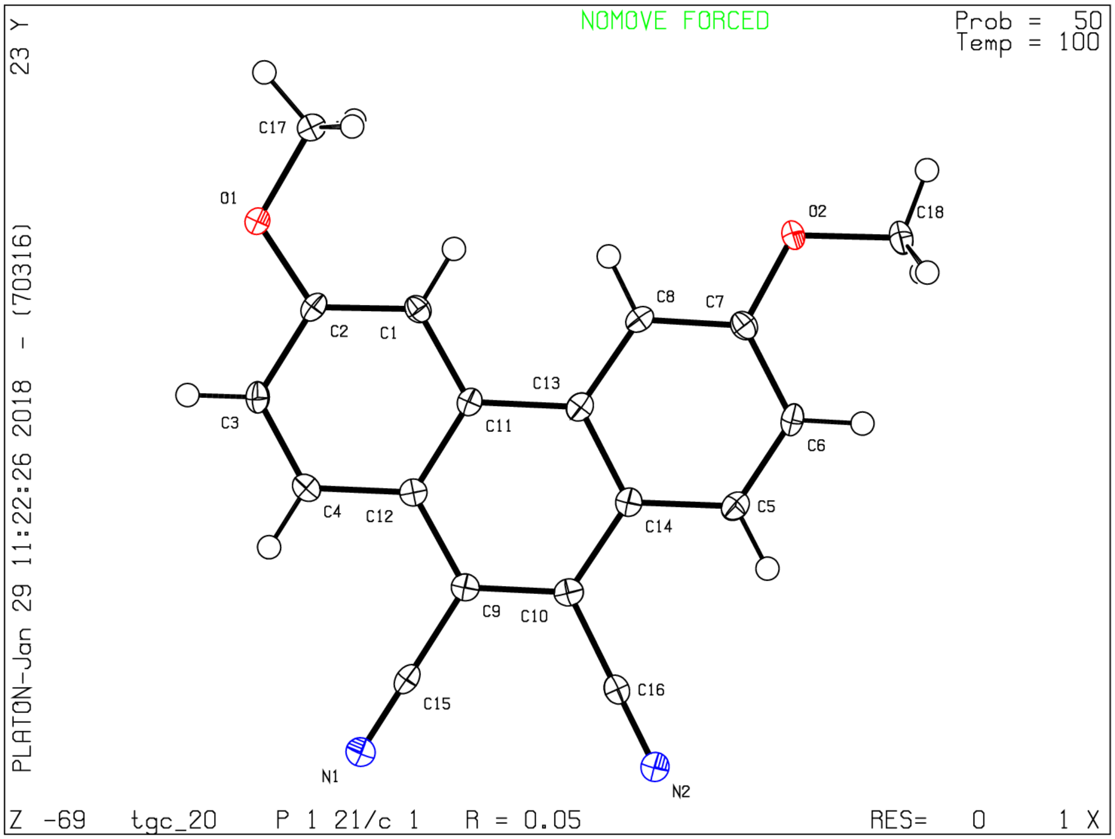

Supplement: Multimedia component 1 [file mmc1.zip › checkcif_7_ССDC 1820117.pdf]
